# Xanthomes éruptifs annonçant une pancréatite aigue grave

**DOI:** 10.11604/pamj.2014.17.225.2521

**Published:** 2014-03-24

**Authors:** Mariem Bounouar, Fatimazahra Mernissi

**Affiliations:** 1 Service de Dermatologie, CHU Hassan II, Université Sidi Mohammed Ben abdellah, Fès, Maroc

**Keywords:** Xanthomes éruptifs, pancréatite, cholécystite, eruptive xanthomas, pancreatitis, cholecystitis

## Image en medicine

Nous rapportons le cas d'une patiente de 34 ans, qui a comme antécédents une cholécystite et une pancréatite il y a 4 ans. Elle a présenté depuis 1 mois et demi une éruption papuleuse prurigineuse généralisée. L'examen dermatologique a objectivé de multiples petites papules jaunâtres entourées par endroits d'un halo inflammatoire, disséminées sur le tronc et les membres. L’étude histologique a montré un amas d'histiocytes spumeux, parfois péri vasculaires, au niveau du derme. L’étude immuno-histochimique a montré un marquage positif par les anticorps anti CD68, CD 163 et négatif pour la protéine S100 et CD1a. L’évolution était marquée par l'installation de douleurs abdominales intenses 8 semaines après le début de l’éruption cutanée; le bilan biologique a montré une hypertriglycéridémie majeure à 20,18 g/l; le scanner abdominal a révélé une pancréatite stade E. Nous avons donc conclu au diagnostic de xanthomes éruptifs révélant une hypertriglycéridémie majeure compliquée d'une pancréatite aigue grave. La patiente a été prise en charge en milieu de réanimation avec bonne évolution clinique. Les xanthomes éruptifs représentent des pseudotumeurs bénignes en rapport avec un trouble du métabolisme des lipoprotéines avec une hypertriglycéridémie et risque de pancréatite. Le diagnostic repose sur la confrontation anatomo-clinique. Leur traitement est purement étiologique, se basant sur le contrôle du trouble lipidique. Ils doivent être considérés comme un signal d'alerte incitant à dépister le trouble métabolique en cause et le corriger afin d’éviter d’éventuelles complications.

**Figure 1:**
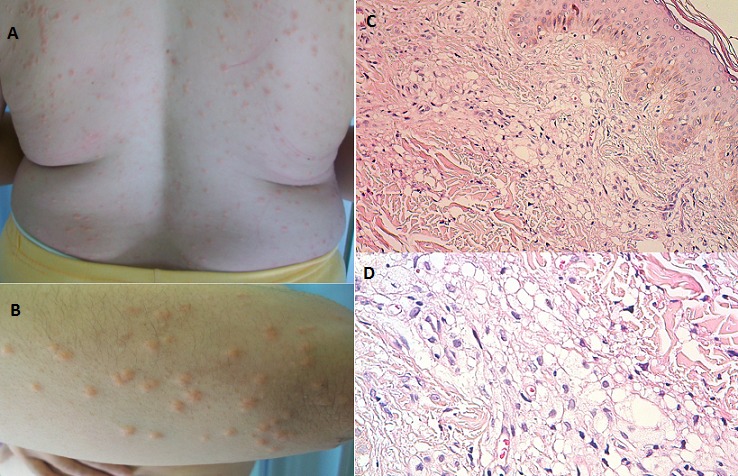
A et B) Papules jaunâtres sur le tronc et les membres. C et D) Aspect histologique montrant un amas d'histiocytes spumeux dermiques (Coloration Hématoxyline-Eosine)

